# Can the digestible indispensable amino acid score methodology decrease protein malnutrition

**DOI:** 10.1093/af/vfz038

**Published:** 2019-09-28

**Authors:** Hannah M Bailey, Hans H Stein

**Affiliations:** Division of Nutritional Sciences, University of Illinois, Urbana, IL

**Keywords:** amino acids, animal protein, digestible indispensable amino acid scores, pig model, plant protein, protein quality

**Implications**

- The new system for estimating protein quality of human foods, which is called “Digestible Indispensable Amino Acid Score” or DIAAS, allows for calculation of the amino acid quality of food proteins that are based on ileal digestibility rather than total tract digestibility and values for each amino acid may be calculated.
- By recognizing the pig as an appropriate model for determining DIAAS values in human food proteins, a procedure for the standardized measurement of DIAAS values in a large number of food proteins has been established.
- Because digestibility values for amino acids in individual food proteins are additive in mixed meals, DIAAS values for mixed meals may be calculated. By comparing DIAAS values of mixed meals to the requirements for digestible indispensable amino acid, the amino adequacy of the meal may be calculated.
- Animal proteins such as meat and milk have greater DIAAS values than plant proteins, but by complementing plant proteins with low DIAAS values with animal proteins with greater DIAAS values, balanced meals that are adequate in all amino acids can be provided.

## Introduction

The characterization of proteins based on their nutritional value and optimization in human diets has been a lasting discussion over the years. Research focusing on protein malnutrition was largely conducted after the identification of kwashiorkor and the realization that many children globally are suffering from subclinical protein malnutrition. To address protein malnutrition, the composition and digestibility of proteins must be determined. Proteins consist of individual amino acids and the sequence and digestibility of each AA varies among proteins. The Food and Agriculture Organization of the United Nations (FAO) has developed methods to evaluate the protein quality of food items and, in 2011, the Digestible Indispensable Amino Acid Score (DIAAS) was recommended as the successor to their previous method: Protein Digestibility Corrected Amino Acid Score (PDCAAS). In the new DIAAS system, each indispensable amino acid is recognized as an individual nutrient and use of the DIAAS system offers a number of advantages in preparation of meals that are adequate in amino acids.

## Indispensable Amino Acid Malnutrition

An estimated 815 million people globally are affected by undernutrition with one in four children under the age of 5 suffering from chronic undernutrition (FAO et al., 2017). The cost of undernutrition is approximately 2.1 trillion USD per year (FAO, 2014). Undernutrition is a global issue; however, sub-Saharan Africa and Southern Asia are among the countries with the greatest prevalence of child chronic malnutrition (FAO et al., 2017). These regions heavily rely on diets composed of cereal grains, such as sorghum, wheat, rice, or maize, which are limiting in indispensable amino acids such as lysine (Cervantes-Pahm et al., 2014; Shaheen et al., 2016; FAO et al., 2017; Abelilla et al., 2018). Cereal grains also undergo a variety of processing methods prior to consumption, which may decrease protein and amino acid digestibility (Duodu et al., 2002; FAO, 2013).

In 1933, a severe disease related to protein deficiency was identified by Cicely Williams: kwashiorkor (Williams, 1933). Kwashiorkor is nearly exclusive to children 1 to 2 yr of age and is characterized by general edema, swollen abdomens, and flaky skin (Williams, 1933). In addition, it was noted that kwashiorkor was mainly associated with children being weaned on maize-based diets (Williams, 1933), which are limiting in lysine (Cervantes-Pahm et al., 2014). Kwashiorkor is a very severe form of protein deficiency; however, there are many children suffering from subclinical protein malnutrition (Semba, 2016).

Around 1970, the study of undernourished children shifted from focusing on protein and amino acids to energy and micronutrients under the assumption that the majority of children are consuming adequate amounts of protein (Semba et al., 2016). However, protein and amino acid deficiencies continued to be addressed by researchers and in 2016, a relationship was identified between chronic undernutrition of children (or stunted children) and low serum levels of all nine indispensable amino acids (Semba et al., 2016). In addition, Ghosh et al. (2012) calculated the risk of protein inadequacy in different countries using estimates of total protein and utilizable (digestible) protein. The risk of protein inadequacy ranged from 0.7% in North America to 37.2% in East and South Africa (Ghosh et al., 2012). These data contributed to reemphasize the need for providing meals that are adequate in all indispensable amino acids. However, to provide meals that are adequate in all indispensable amino acids, the quality of the protein in each food item needs to be determined and mixed meals need to be adjusted for protein quality by complementing low-quality proteins with higher quality proteins. The quality of protein in a food item is primarily determined by the concentration of the first limiting indispensable amino acid and the prececal—or ileal—digestibility of all indispensable amino acids.

## Diaas Principles

**Figure 1.**
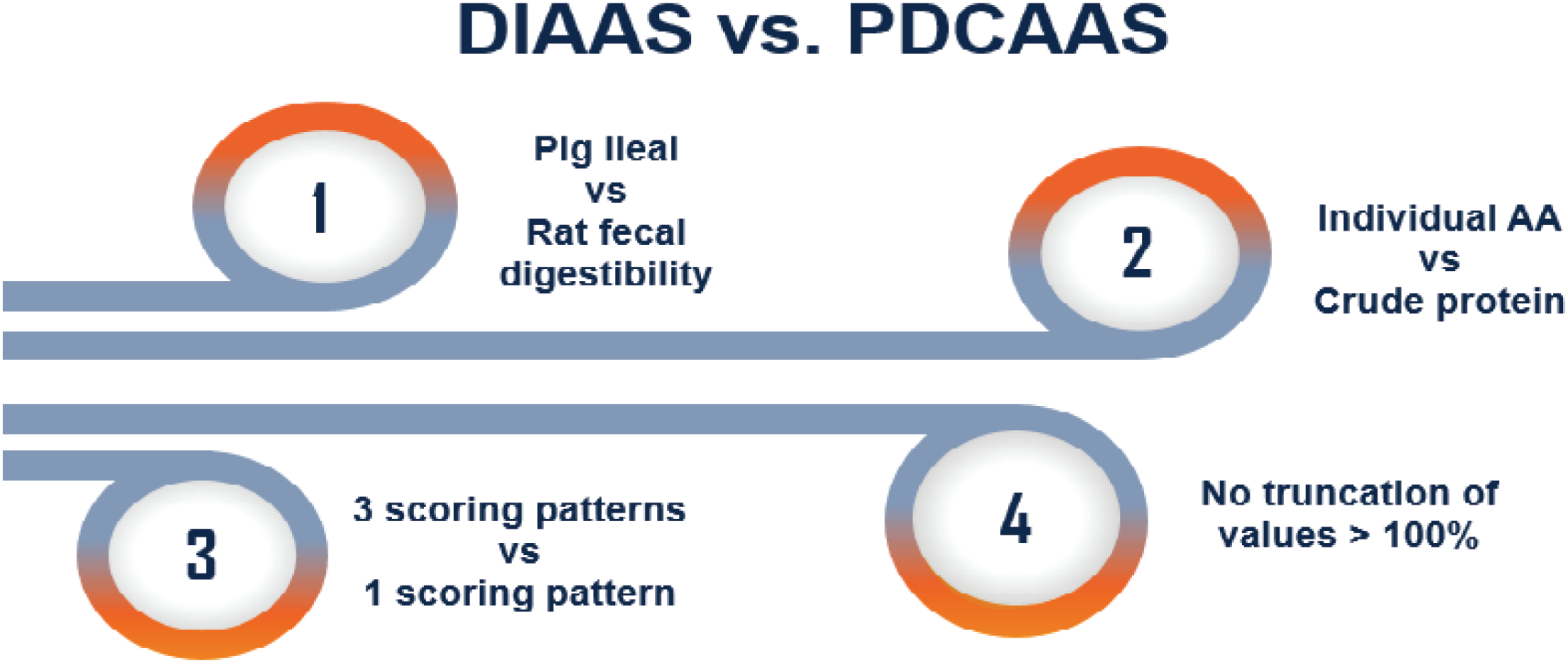
Differences between the protein digestibility corrected amino acid score (PDCAAS) system and the digestible indispensable amino acid score (DIAAS) system.

The DIAAS methodology was developed to overcome multiple limitations of the previous method, PDCAAS, used to evaluate protein quality (Figure 1). A number of reviews outlining the limitations of the PDCAAS method have been published (WHO, 2007; Boye et al., 2012; Gilani, 2012; Schaafsma, 2012; FAO, 2013); however, PDCAAS will continue to be used until a sufficient database of ileal digestibility and DIAAS values are generated for commonly consumed human foods. There is therefore a need to generate DIAAS values for food proteins commonly consumed in different areas of the world.

### Pig vs. rat animal model

The human is considered the best subject to determine ileal amino acids digestibility values for humans; however, the FAO has recognized that when values cannot be determined in the human, the growing pig is the more appropriate animal model compared with the growing rat (FAO, 2013). The pig is superior to the rat because it is a meal-eating species, similar to humans (Gilani, 2012). In addition, the gastrointestinal anatomy of the pig is similar to that of humans, and the physiology and metabolism response to nutrients ingested by pigs are comparable to humans (Gilani, 2012). In addition, the rat has a unique requirement for sulfur containing amino acids because of the high concentration of cysteine in the fur, but this is not the case for the pig (Deglaire and Moughan, 2012). It is, therefore, recommended that DIAAS values of food proteins are determined from ileal digestibility values of amino acids that are obtained in the growing pig if values cannot be determined in humans (FAO, 2013).

### Amino acid vs. protein digestibility

The DIAAS methodology determines the digestibility of each individual amino acid. This is arguably the most significant change in the transition from PDCAAS to DIAAS because the potential differences of individual amino acid digestibility are now considered. This is especially important for food items that have been processed or heated, as well as for food items that have a high concentration of antinutritional factors. Processing, heating, and antinutritional factors can decrease the bioavailability or digestibility of different amino acids (Moughan, 2003; Gilani et al., 2012). As an example, the epsilon amino group of lysine is very susceptible to reacting with a reducing sugar at high temperatures and the associated Maillard reactions will reduce the concentration as well as the digestibility of lysine (Moughan, 2003). This is extremely relevant when determining the protein quality of diets based on a mixture of food proteins. For example, a mixed diet based on cereal grains may appear to meet the crude protein requirement for an age group; however, certain amino acids may not be present in adequate amounts and diets based primarily on cereal grains do usually not meet the requirement for digestible lysine. Therefore, it is important to treat each individual amino acid as a single nutrient when evaluating the digestibility and quality of a protein.

### Ileal vs. total tract digestibility

Amino acid absorption takes place entirely in the small intestine of pigs, humans, and all other animals (Moughan, 2003; Stein et al., 2007). However, the PDCAAS methodology determined protein digestibility from fecal samples (FAO, 1991). Proteins synthesized by microbes and other nondietary proteins also end up in fecal contents, which will result in an overestimation of PDCAAS for low-quality proteins (Mathai et al., 2017). Therefore, the DIAAS methodology recommends amino acid digestibility be measured at the end of the small intestine (FAO, 2013). The most common method to measure this in pigs is by surgically inserting a T-cannula into the end of the small intestine (Stein et al., 1998). This method is effective and is being used across the globe to determine the ileal digestibility of amino acids associated with many foods and food ingredients. By collecting digesta at the end of the small intestine, it is also possible to directly calculate the small intestinal digestibility of each individual amino acid and, therefore, apply unique digestibility values to each amino acid. In contrast, fecal collections, as used in the PDCAAS methodology, only allow calculation of the digestibility of crude protein. This value is then applied to all amino acids assuming that the digestibility of all amino acids is the same, which has been demonstrated not to be a correct assumption (Mathai et al., 2017).

### Values greater than 100 vs. truncation

The PDCAAS methodology requires values greater than 100% to be truncated to 100% (FAO, 2013), due to the assumption that consuming amino acids at a concentration greater than the human amino acids requirement does not provide addition nutritional benefit (Schaafsma, 2012). However, this approach fails to recognize the ability of high-quality proteins to complement low-quality proteins in mixed meals. Humans almost always consume a combination of ingredients during each meal and under such conditions, high-quality proteins are used to balance low-quality proteins to provide a complete meal that is nutritionally adequate in all amino acids.

**Figure 2.**
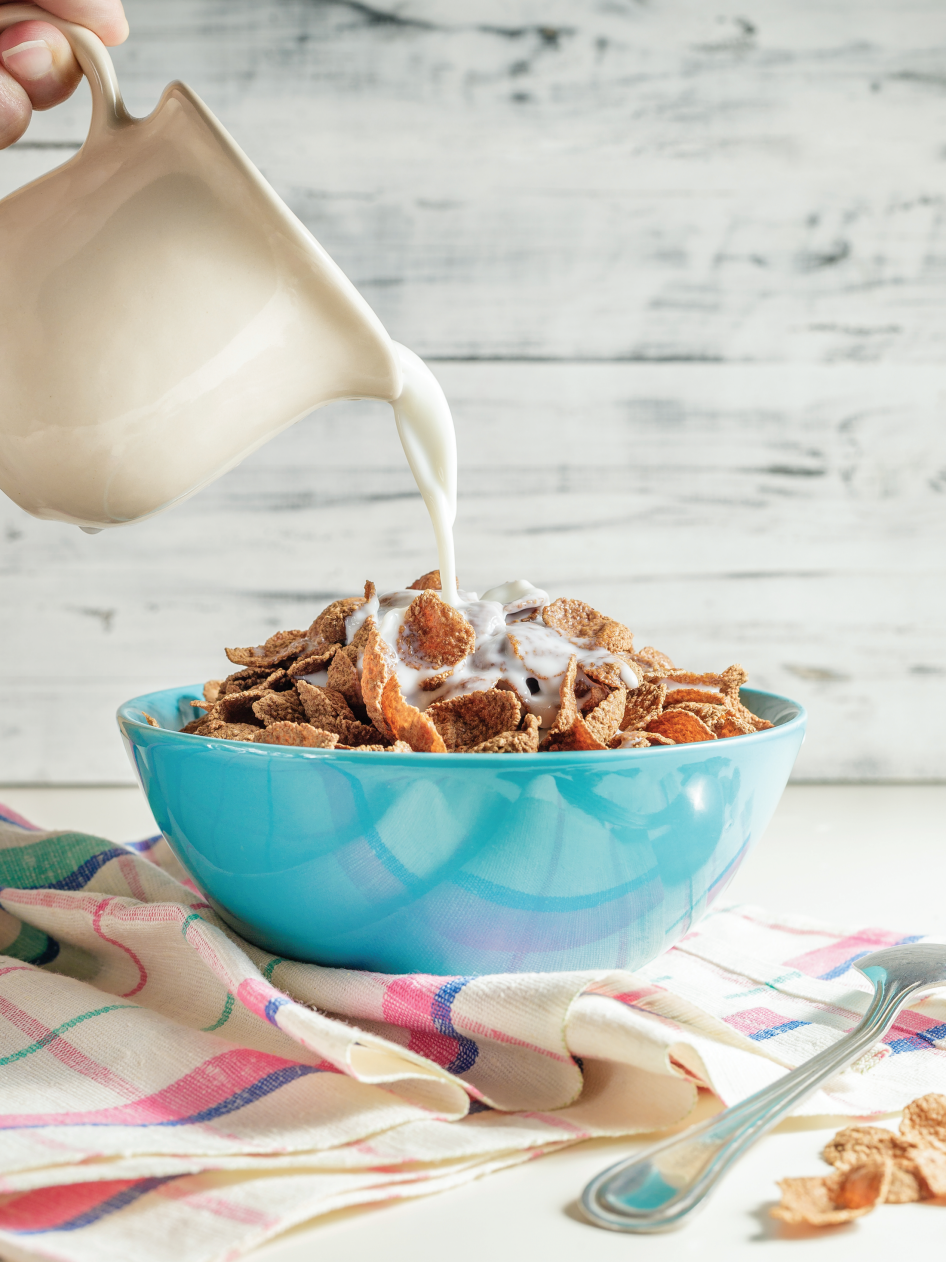
The calculated digestible indispensable amino acid score (DIAAS) value of a mixed meal of 60% milk and 40% wheat based breakfast cereal is 107, demonstrating the ability of milk to complement wheat resulting in a balanced meal that provides 100% of human AA requirements.

Therefore, the DIAAS methodology does not truncate values at 100% and an example of the benefit of this is a mixed meal of milk and wheat (Figure 2). Wheat has a DIAAS value of 45 (Mathai et al., 2017); however, when wheat is processed in the form of a breakfast cereal, it may only have a DIAAS value of 1 (Rutherfurd et al., 2015). In contrast, milk has a DIAAS value of 118 (Rutherfurd et al., 2015). The calculated DIAAS value of a mixed meal of 60% milk and 40% breakfast cereal is 107 (Rutherfurd et al., 2015), demonstrating the ability of milk to complement wheat resulting in a balanced meal that meet the requirement for all indispensable AA. Likewise, it was recently demonstrated that milk and eggs are efficient in complementing low-quality plant proteins to improve the DIAAS value (Shivakumar et al., 2019). Although legumes generally have a greater DIAAS value than cereal grains, they are limiting in methionine and may contain antinutritional factors that often reduce the absorption of amino acids or micronutrients (Rutherfurd et al., 2015, Shivakumar et al., 2019). Consequently, animal proteins are more effective in increasing the protein quality of mixed meals and meeting human amino acid requirements than proteins from legumes.

## Protein Claims

Based on the FAO recommendation, DIAAS values can be calculated for three age groups; 1) infants from birth to 6 mo, 2) children from 6 mo to 3 yr, and 3) children older than 3 yr, adolescents, and adults (FAO, 2013). Upon determination of DIAAS for a specific food item and age group, a protein claim can be made and added to its food label (FAO, 2013). This claim is based on the determined DIAAS value and, therefore, takes into account the bioavailability of amino acids and amino acid concentrations relative to human amino acids requirements. If a food item has a DIAAS value greater than 100, it can be considered an “excellent” quality protein source for the specific age group. A food item can be considered a “good” quality protein source if the DIAAS value is between 75 and 99. However, a food item with a DIAAS value less than 75 cannot have a claim made for protein (FAO, 2013). Generally, animal proteins (i.e., dairy, eggs, and meat) are considered “excellent” quality proteins with DIAAS values greater than 100 (Table 1; Rutherfurd et al., 2015; Mathai et al., 2017; Hodgkinson et al., 2018). In contrast, plant proteins and cereal grains generally have DIAAS values that are less than 75, with the exception of soy protein that usually has a DIAAS value between 75 and 100 and oats with a DIAAS value around 75 (Cervantes-Pahm et al., 2014; Rutherfurd et al., 2015; Mathai et al., 2017; Abelilla et al., 2018; Han et al., 2019).

**Table 1.** Digestible indispensable amino acid scores (DIAAS) and first-limiting amino acid (AA) in parentheses determined for human foods using the pig or rat model

		Reference protein pattern			
	Animal model	Infants (0 to 6 mo)	Young children (6 months to 3 yr)	Older children, adolescents, and adults	Reference
Cereal grains					
Corn, yellow dent, raw	Pig	–	–	48 (lysine)	Cervantes-Pahm et al., 2014
Millet, cooked	Rat	–	10 (lysine)	–	Han et al., 2019
Oats, rolled, cooked	Rat	–	54 (lysine)	–	Rutherfurd et al., 2015
Rice, polished, cooked	Rat	–	37 (lysine)	–	Han et al., 2019
Sorghum, raw	Pig	–	–	29 (lysine)	Cervantes-Pahm et al., 2014
Wheat, raw	Pig	37 (lysine)	45 (lysine)	54 (lysine)	Mathai et al., 2017
Wheat, whole, cooked	Rat	–	20 (lysine)	–	Han et al., 2019
Plant proteins					
Kidney beans, cooked	Rat	–	59 (SAA)	–	Rutherfurd et al., 2015
Peas, cooked	Rat	–	58 (SAA)	–	Rutherfurd et al., 2015
Soy protein isolate	Pig	68 (SAA)	84 (SAA)	98 (SAA)	Mathai et al., 2017
Dairy proteins					
Milk protein concentrate	Pig	85 (tryptophan)	120 (SAA)	141 (SAA)	Mathai et al., 2017
Skimmed milk powder	Pig	81 (threonine)	105 (SAA)	123 (SAA)	Mathai et al., 2017
Whey protein concentrate	Pig	71 (AAA)	107 (histidine)	133 (histidine)	Mathai et al., 2017
Meat proteins					
Beef, raw	Pig	–	97 (valine)	–	Hodgkinson et al., 2018
Beef, boiled, 71 °C	Pig	–	99 (valine)	–	Hodgkinson et al., 2018
Beef, roasted, 71 °C	Pig	–	91 (valine)	–	Hodgkinson et al., 2018

First-limiting amino acid (AA) is in parentheses. Unreported DIAAS values for certain reference patterns are noted by “–.” SAA = sulfur AA; AAA = aromatic AA.

**Figure 3.**
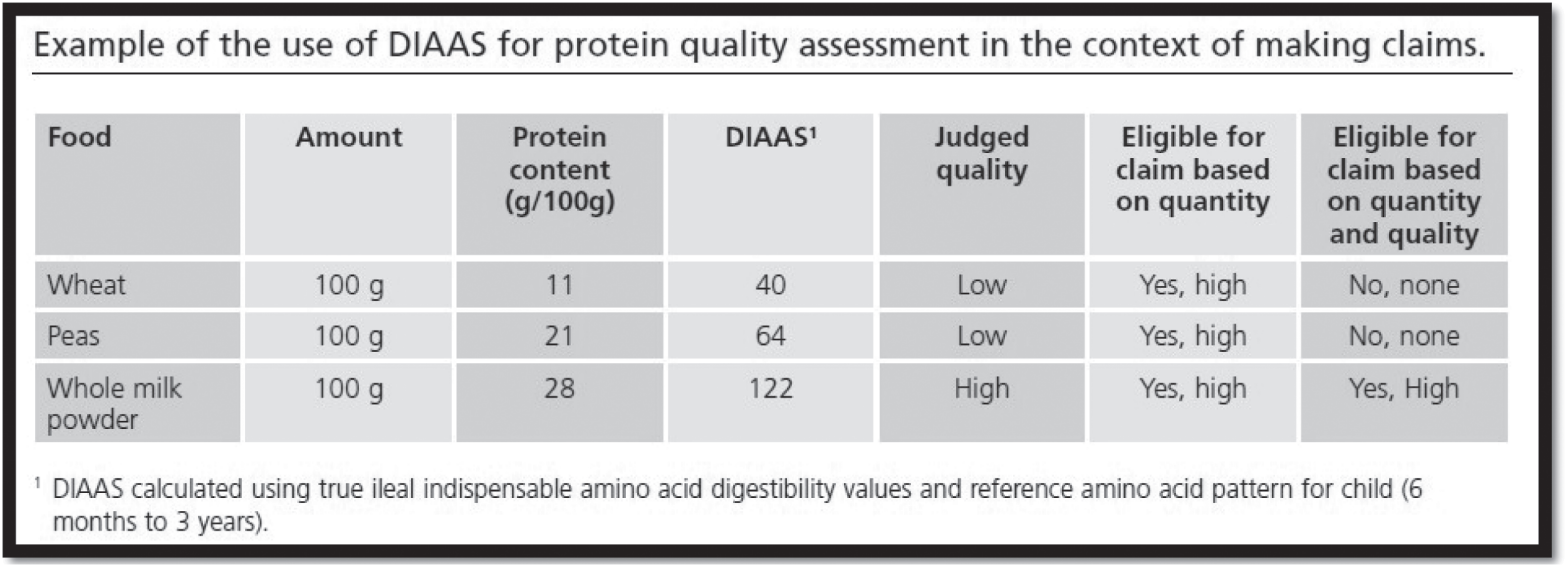
Example of the use of digestible indispensable amino acid score (DIAAS) values in making protein quality claims and how it differs from protein quantity claims. Adopted from FAO, 2013.

A DIAAS value greater than 100 indicates that if the food item is consumed in an amount equivalent to the estimated average requirement for protein (i.e., 0.66 g kg^−1^ d^−1^; IOM, 2002/2005), 100% or more of the human amino acid requirements will be met for the day. However, the protein content listed on the food label is not indicative of the quality of amino acids in the food (Figure 3). For example, peas may have a high quantity of protein, but with a DIAAS value of approximately 64 it has a low quality, whereas milk has both a high quantity of protein and high quality of amino acids with a DIAAS of 122. As a consequence, an individual would have to consume more than twice as much pea protein compared with milk protein to meet the human amino acid requirements. This illustrates that both the quantity and the quality of protein are important when using DIAAS to formulate meals adequate in all amino acids.

## Protein Complementation

**Figure 4.**
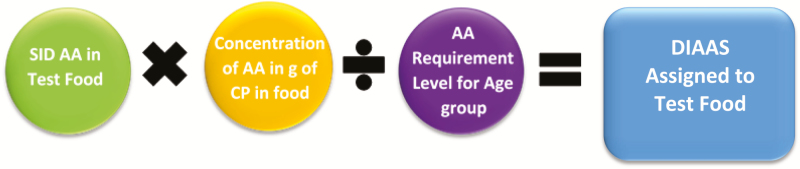
Steps in calculating digestible indispensable amino acid score (DIAAS) values. First, the standardized ileal digestibility (SID) of amino acid (AA) in the test food is determined in the human, pig, or rat. Then, this value (SID of AA in Test Food) is multiplied by the analyzed concentration (mg) of the same AA in a g of crude protein (CP) for the test food. Lastly, the calculated mg SID of each AA per gram of CP is divided by the same AA requirement level for a specific age group. This results in a calculated digestible indispensable AA (DIAA) reference value for each AA and the AA in least concentration is the DIAAS value assigned to the test food.

The DIAAS methodology enables the determination of complementary proteins (FAO, 2013). Values for DIAAS in individual ingredients are calculated by first determining the standardized ileal digestibility of each amino acid in a food protein. This value is then multiplied by the concentration of that amino acid in the protein to calculate mg digestible amino acid per g protein. A digestible indispensable amino acid reference value is then calculated for each amino acid by dividing the concentration of digestible amino acid by the reference value for a specific age group. The DIAAS value is subsequently determined as the least value among the digestible indispensable amino acid values for the digestible indispensable amino acids (Figure 4).

In addition, the protein quality of mixed meals can be determined due to the additivity of DIAAS values calculated for ingredients (FAO, 2013). These two aspects of the DIAAS methodology are useful in evaluating and recommending diets consumed in developing countries that may not be adequate in amino acids. For example, polished rice (DIAAS of approximately 60) is limiting in lysine, but has a high concentration of digestible sulfur amino acids. As a consequence, rice may complement peas (DIAAS of approximately 58) that are limiting in digestible sulfur amino acids and high in lysine (Rutherfurd et al., 2015). The combination of these two ingredients may provide a balanced amino acid pattern in a mixed meal. Another example is how meat, a good-quality protein with a DIAAS of approximately 99 (Hodgkinson et al., 2018), can complement wheat, a cereal grain limiting in lysine with a DIAAS of approximately 54 (Mathai et al., 2017), to provide all amino acids that are greater than or equal to human AA requirements. Table 1 summarizes DIAAS values for common ingredients that have been published and determined in the growing rat or the growing pig. Han et al. (2019) determined DIAAS values in cereal grains commonly produced in China. However, DIAAS values need to be determined in ingredients commonly consumed in developing countries to accurately determine the protein quality of diets consumed. This will make it possible to make recommendations on how meals that are balanced in amino acids can be prepared, which will contribute to reducing the prevalence of protein malnutrition.

## Summary

Protein undernutrition is a serious global problem that results in stunted growth, disease, and premature death in millions of people annually. Protein deficiency is caused by a deficiency in specific indispensable amino acids and efforts to alleviate protein malnutrition, therefore, need to focus on the provision of adequate quantities of digestible indispensable amino acids. The digestibility of amino acids is most correctly determined at the end of the small intestine, and digestibility values obtained using this approach are termed “ileal digestibility values.” The total protein provision to an individual is based on the sum of digestible indispensable amino acids consumed in a meal. The protein quality of a single ingredient is less important than the quality of the mixed meal that is consumed. To assist in calculating the protein quality of meals for humans, the FAO recommends that a system called the “Digestible Indispensable Amino Acid Score” or DIAAS be used. This system is based on the ileal digestibility of each amino acid in individual protein foods, but allows for the calculation of the quality of a meal consisting of a number of protein foods. The system can then be used to assess the quality of total protein intake consumed as a meal. Comparison of this value to the requirements for indispensable amino acids by humans can be used to estimate an individual’s protein status.

## About the Authors

**Hannah Bailey** is a PhD student studying monogastric nutrition in the Division of Nutritional Science at the University of Illinois, Urbana–Champaign. She has conducted research to determine DIAAS values for a number of processed and nonprocessed meat products. Hannah received her BS in Animal Science from Iowa State University and worked at Cargill-Provimi for 1 yr as an External Swine Formulator prior to arriving at the University of Illinois.

**Hans H. Stein** is a professor of Nutrition at the University of Illinois, USA. His research focuses on determining nutrient and energy digestibility in humans and pigs. He has also conducted multiple experiments to determine the protein value of human foods. Dr. Stein has received a number of awards for his research, extension, and teaching activities.

## References

[CIT0001] Abelilla J. J., Liu Y., and Stein H. H. 2018 Digestible indispensable amino acid score (DIAAS) and protein digestibility corrected amino acid score (PDCAAS) in oat protein concentrate measured in 20- to 30-kilogram pigs. J. Sci. Food Agric. 98:410–414. doi:10.1002/jsfa.8457 28573795

[CIT0002] Boye J., Wijesinha-Bettoni R., and Burlingame B. 2012 Protein quality evaluation twenty years after the introduction of the protein digestibility corrected amino acid score method. Br. J. Nutr. 108 (Suppl 2):S183–S211. doi:10.1017/S0007114512002309 23107529

[CIT0003] Cervantes-Pahm S. K., Liu Y., and Stein H. H. 2014 Digestible indispensable amino acid score and digestible amino acids in eight cereal grains. Br. J. Nutr. 111:1663–1672. doi:10.1017/S0007114513004273 24480298

[CIT0004] Deglaire A., and Moughan P. J. 2012 Animal models for determining amino acid digestibility in humans - a review. Br. J. Nutr. 108 (Suppl 2):S273–S281. doi:10.1017/S0007114512002346 23107538

[CIT0005] Duodu K. G., Nunes A., Delgadillo I., Parker M. L., Mills E. N. C., Belton P. S., and Taylor J. R. N. 2002 Effect of grain structure and cooking on sorghum and maize in vitro protein digestibility. J. Cereal Sci. 35:161–174. doi:10.1006/jcrs.2001.0411

[CIT0006] FAO, IFAD, UNICEF, WFP, and WHO 2017 The state of food security and nutrition in the world 2017. Building resilience for peace and food security. Rome: Food and Agriculture Organization of the United Nations Available from http://www.fao.org/3/a-i7695e.pdf (accessed June 2019).

[CIT0007] Food and Agriculture Organization of the United Nations (FAO) 1991 Protein quality evaluation. Report of a Joint FAO/WHO Expert Consultation. FAO food and nutrition paper 51. Rome: Food and Agriculture Organization of the United Nations.

[CIT0008] Food and Agriculture Organization of the United Nations (FAO) 2013 Dietary protein quality evaluation in human nutrition. Report of an FAO expert group. FAO food and nutrition paper 92. Rome: Food and Agriculture Organization of the United Nations.

[CIT0009] Food and Agriculture Organization of the United Nations (FAO) 2014 Understanding the true cost of malnutrition Rome: Food and Agriculture Organization of the United Nations Available from http://www.fao.org/zhc/detail-events/en/c/238389/ (accessed June 2019).

[CIT0010] Ghosh S., Suri D., and Uauy R. 2012 Assessment of protein adequacy in developing countries: quality matters. Br. J. Nutr. 108 (Suppl 2):S77–S87. doi:10.1017/S0007114512002577 23107551

[CIT0011] Gilani G. S 2012 Background on international activities on protein quality assessment of foods. Br. J. Nutr. 108 (Suppl 2):S168–S182. doi:10.1017/S0007114512002383 23107528

[CIT0012] Gilani G. S., Xiao C. W., and Cockell K. A. 2012 Impact of antinutritional factors in food proteins on the digestibility of protein and the bioavailability of amino acids and on protein quality. Br. J. Nutr. 108:S315-S332. doi:10.1017/S000711451200371 23107545

[CIT0013] Han F., Han F., Wang Y., Fan L., Song G., Chen X., Jiang P., Miao H., and Han Y. 2019 Digestible indispensable amino acid scores of nine cooked cereal grains. Br. J. Nutr. 121:30–41. doi:10.1017/S0007114518003033 30396372

[CIT0014] Hodgkinson S. M., Montoya C. A., Scholten P. T., Rutherfurd S. M., and Moughan P. J. 2018 Cooking conditions affect the true ileal digestible amino acid content and Digestible Indispensable Amino Acid Score (DIAAS) of Bovine meat as determined in pigs. J. Nutr. 148:1564–1569. doi:10.1093/jn/nxy153 30204886

[CIT0015] Institute of Medicine of the National Academies (IOM) 2002/2005 Dietary reference intakes for energy, carbohydrates, fiber, fat, protein and amino acids. Washington, DC: The National Academies Press.

[CIT0016] Mathai J. K., Liu Y., and Stein H. H. 2017 Values for digestible indispensable amino acid scores (DIAAS) for some dairy and plant proteins may better describe protein quality than values calculated using the concept for protein digestibility-corrected amino acid scores (PDCAAS). Br. J. Nutr. 117:490–499. doi:10.1017/S0007114517000125 28382889

[CIT0017] Moughan P. J 2003 Amino acid availability: aspects of chemical analysis and bioassay methodology. Nutr. Res. Rev. 16:127–141. doi:10.1079/NRR200365 19087386

[CIT0018] Rutherfurd S. M., Fanning A. C., Miller B. J., and Moughan P. J. 2015 Protein digestibility-corrected amino acid scores and digestible indispensable amino acid scores differentially describe protein quality in growing male rats. J. Nutr. 145:372–379. doi:10.3945/jn.114.195438 25644361

[CIT0019] Schaafsma G 2012 Advantages and limitations of the protein digestibility-corrected amino acid score (PDCAAS) as a method for evaluating protein quality in human diets. Br. J. Nutr. 108 (Suppl 2):S333–S336. doi:10.1017/S0007114512002541 23107546

[CIT0020] Semba R. D 2016 The rise and fall of protein malnutrition in global health. Ann. Nutr. Metab. 69:79–88. doi:10.1159/000449175 27576545 PMC5114156

[CIT0021] Semba R. D., Shardell M., Sakr Ashour F. A., Moaddel R., Trehan I., Maleta K. M., Ordiz M. I., Kraemer K., Khadeer M. A., Ferrucci L., et al 2016 Child stunting is associated with low circulating essential amino acids. Ebiomedicine. 6:246–252. doi:10.1016/j.ebiom.2016.02.030 27211567 PMC4856740

[CIT0022] Shaheen N., Islam S., Munmun S., Mohiduzzaman M., and Longvah T. 2016 Amino acid profiles and digestible indispensable amino acid scores of proteins from the prioritized key foods in Bangladesh. Food Chem. 213:83–89. doi:10.1016/j.foodchem.2016.06.057 27451158

[CIT0023] Shivakumar N., Kashyap S., Kishore S., Thomas T., Varkey A., Devi S., Preston T., Jahoor F., Sheshshayee M. S., and Kurpad A. V. 2019 Protein-quality evaluation of complementary foods in Indian children. Am. J. Clin. Nutr. 109:1319–1327. doi:10.1093/ajcn/nqy265 30920607 PMC6499502

[CIT0024] Stein H. H., Sève B., Fuller M. F., Moughan P. J., and de Lange C. F.; Committee on Terminology to Report AA Bioavailability and Digestibility 2007 Invited review: amino acid bioavailability and digestibility in pig feed ingredients: terminology and application. J. Anim. Sci. 85:172–180. doi:10.2527/jas.2005-742 17179553

[CIT0025] Stein H. H., Shipley C. F., and Easter R. A. 1998 Technical note: a technique for inserting a T-cannula into the distal ileum of pregnant sows. J. Anim. Sci. 76:1433–1436. doi:10.2527/1998.7651433x 9621950

[CIT0026] Williams C. D 1933 A nutritional disease of childhood associated with a maize diet. Arch. Dis. Child. 8:423–433. doi:10.1136/adc.8.48.423 21031941 PMC1975318

[CIT0027] World Health Organization (WHO) 2007 Protein and amino acid requirements in human nutrition. Report of a joint FAO/WHO/UNU expert consultation. WHO technical reports series no. 935. Geneva (Switzerland): WHO.

